# Localization of hyperpolarization-activated cyclic nucleotide-gated channels in the vertebrate retinas across species and their physiological roles

**DOI:** 10.3389/fnana.2024.1385932

**Published:** 2024-03-18

**Authors:** Daniel Kim, Hyeonhee Roh, Hyung-Min Lee, Sang Jeong Kim, Maesoon Im

**Affiliations:** ^1^Brain Science Institute, Korea Institute of Science and Technology (KIST), Seoul, Republic of Korea; ^2^Department of Biomedical Sciences, College of Medicine, Seoul National University (SNU), Seoul, Republic of Korea; ^3^School of Electrical Engineering, College of Engineering, Korea University, Seoul, Republic of Korea; ^4^Division of Bio-Medical Science & Technology, KIST School, University of Science & Technology (UST), Seoul, Republic of Korea; ^5^KHU-KIST Department of Converging Science and Technology, Kyung Hee University, Seoul, Republic of Korea

**Keywords:** HCN channels, central nervous system (CNS), retina, localization across species, retinal degeneration

## Abstract

Transmembrane proteins known as hyperpolarization-activated cyclic nucleotide-gated (HCN) channels control the movement of Na^+^ and K^+^ ions across cellular membranes. HCN channels are known to be involved in crucial physiological functions in regulating neuronal excitability and rhythmicity, and pacemaker activity in the heart. Although HCN channels have been relatively well investigated in the brain, their distribution and function in the retina have received less attention, remaining their physiological roles to be comprehensively understood. Also, because recent studies reported HCN channels have been somewhat linked with the dysfunction of photoreceptors which are affected by retinal diseases, investigating HCN channels in the retina may offer valuable insights into disease mechanisms and potentially contribute to identifying novel therapeutic targets for retinal degenerative disorders. This paper endeavors to summarize the existing literature on the distribution and function of HCN channels reported in the vertebrate retinas of various species and discuss the potential implications for the treatment of retinal diseases. Then, we recapitulate current knowledge regarding the function and regulation of HCN channels, as well as their relevance to various neurological disorders.

## 1 Introduction

Individual neurons of the nervous system express various types of ion channels and distinct constellations of those ion channels determine the unique patterns of activity in each neuron (Goaillard and Marder, 2021), which eventually affect high level physiological functions such as behaviors. Among more than 140 different types of voltage-gated ion channels (Yu et al., 2005), hyperpolarization-activated cyclic nucleotide-gated (HCN) channels have a unique voltage-dependent property compared to other channels such as sodium, potassium, and calcium channels (Goaillard and Marder, 2021). Although the exact molecular mechanism is unknown, HCN channels open in response to hyperpolarizing membrane voltages while other channels mainly open in depolarizing membrane voltages (Flynn and Zagotta, 2018). These channels permit the passage of sodium and potassium ions upon responding to changes in membrane potential (Benarroch, 2013). HCN channels are found in many different areas of the brain as well as in peripheral sensory neurons (Baruscotti et al., 2022). They are principally in charge of producing the hyperpolarization-activated current, Ih, which is crucial in structuring neuronal activities such as neuronal excitability, rhythmic oscillations, synaptic integration, and pacemaker activity (Wahl-Schott and Biel, 2009). Those are achieved by regulating resting membrane potential (RMP), altering synaptic transmission, and directing action potential firing patterns (Biel et al., 2009; Chang et al., 2019).

The regulation of the RMP and the temporal tuning of the potential in photoreceptor cells are functions in which HCN channels actively participate in the retina (Barrow and Wu, 2009). In addition to these neurophysiological functions of HCN channels in the healthy retina, it appears that those channels affect the speed of retinal degeneration (Barrow and Wu, 2009; Della Santina et al., 2012; Schön et al., 2016). For example, a recent study using a mouse model of CNGB1-linked retinitis pigmentosa (RP) revealed the contributory role of HCN1 isoform in the degeneration of photoreceptor cells (Schön et al., 2016). The absence of HCN1 in CNGB1-knockout (K/O) mice resulted in an increased deterioration of photoreceptor cells, underscoring the notion that suppressing HCN channels could exacerbate the progression of RP [Schön et al., 2016; but see Della Santina et al., 2010 for no effect on the speed of retinal degeneration when comparing the retinas of normal mice and those treated with ivabradine, in the *rd10* and wild-type (*wt*) mice]. This result suggests the possible relationship between HCN channel malfunction and retinal disorders. Thus, understanding the specific mechanisms by which HCN channels contribute to these disorders may be helpful for developing targeted therapeutic interventions. Indeed, HCN channels have been regarded as a possible target for therapeutic strategies in various diseases (Postea and Biel, 2011; Sartiani et al., 2015). Thus, modulating the activity of HCN channels through pharmacological agents or gene therapy approaches may offer a potential strategy to restore normal retinal function and mitigate the progression of retinal disorders. However, there are still significant gaps in our knowledge regarding the precise function of HCN channels in different retinal cell types; the comprehensive understanding of underlying molecular mechanisms in related retinal pathologies would help better shape specific therapeutic strategies that can effectively target these channels.

This paper aims to systematically analyze previous literature to summarize the known physiological roles and localization of each HCN isoform across different retinal layers. Additionally, we discuss the potential therapeutic implications of targeting HCN channels and propose future research directions to advance our understanding of these channels and their therapeutic potential in the context of retinal diseases. We also recapitulate the current understanding of the HCN channels in the nervous system (Biel et al., 2009; Wahl-Schott and Biel, 2009; Benarroch, 2013) and pathologies related to their malfunction including epilepsy, neuropathic pain, and Parkinson’s disease, thereby opening the ways for therapeutic approaches targeting these channels.

## 2 The structure of HCN channels and their isoforms

**Figure 1:**
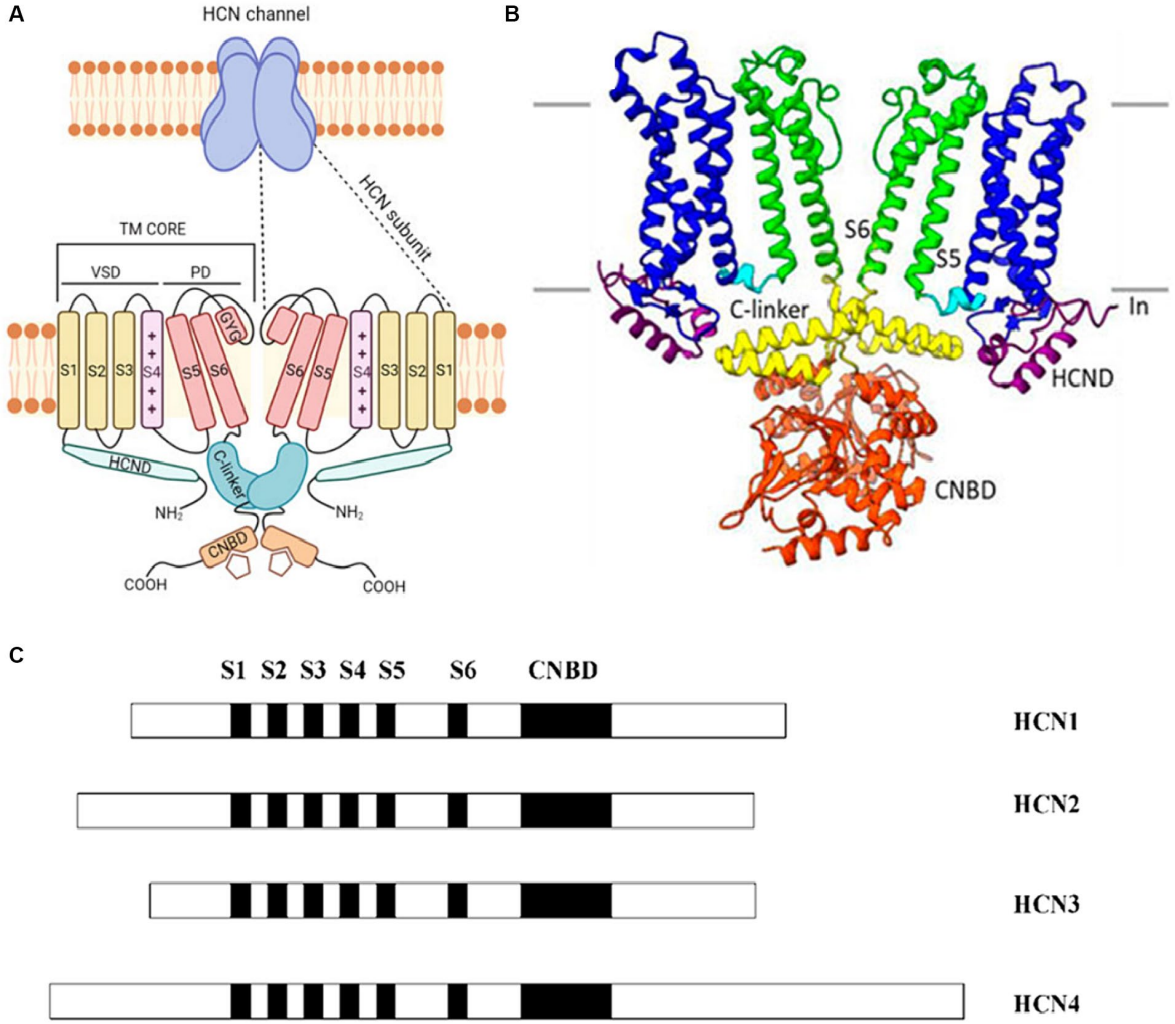
**(A)** A cartoon showing the structure of a HCN channel. HCN channels are tetramers (upper panel). In the lower panel, only two subunits are shown. Each subunit contains the voltage-sensitive (VSD), formed by S1–S3 transmembrane segments (yellow) and by the S4 voltage sensor segment (pink). The pore domain (PD) shown in red is formed by S5-S6 transmembrane segments and the S5-S6 loop. The C-terminus includes the C-linker (green) and the cyclic nucleotide binding domain (CNBD) (orange). The N-terminus contains the HCND (light green). Adapted from Baruscotti et al. (2022) © 2022 European Biophysical Societies’ Association (EBSA). **(B)** The HCN4 CryoEM structure shows a clear representation by exhibiting only two of the four subunits. Each subunit has six transmembrane-spanning domains that are important for voltage sensing (S1–S4, shown in blue) and pore creation (S5–S6, shown in green). The C-linker (shown in yellow) and the CNBD (displayed in orange) are both found in the C-terminus and aid in the response to cyclic nucleotides. Adapted from Peters et al. (2022) © 2022 Peters, Singh, Bankston and Proenza. This is an open-access article distributed under the terms of the Creative Commons Attribution License (CC BY). The use, distribution or reproduction in other forums is permitted, provided the original author(s) and the copyright owner(s) are credited and that the original publication in this journal is cited, in accordance with accepted academic practice. No use, distribution or reproduction is permitted which does not comply with these terms. **(C)** The arrangement of the four known HCN isoforms (1–4) is illustrated, indicating the locations of the six transmembrane segments (S1–S6) and CNBD. Different isoforms of HCN channels share significant structural similarity in their core regions like the transmembrane segments (S) and CNBDs, but exhibit notable variations in the N and C termini. Adapted from Viscomi et al. (2001) Open Access [Attribution 4.0 International (CC BY 4.0)].

Distinguished by their expression patterns and functional properties, the four isoforms of HCN channels (HCN1-4) possess a shared structure composed of six transmembrane segments (S1-S6) and a cyclic nucleotide-binding domain (CNBD) located in the cytoplasmic C-terminus of the protein (Figures 1A,B). Despite sharing a common structure, the HCN isoforms exhibit variations in both their structure and function (Figure 1C). An examination of the sequence structures of different isoforms of HCN channels shows significant similarity in the essential core regions, specifically the transmembrane segment (S) and CNBDs. However, notable variations are observed in the N terminus and the C terminus of these isoforms (Figure 1C). For example, HCN4 has the longest C terminus and HCN3 has the shortest N terminus as compared to other isoforms (Figure 1C).

**Figure 2:**
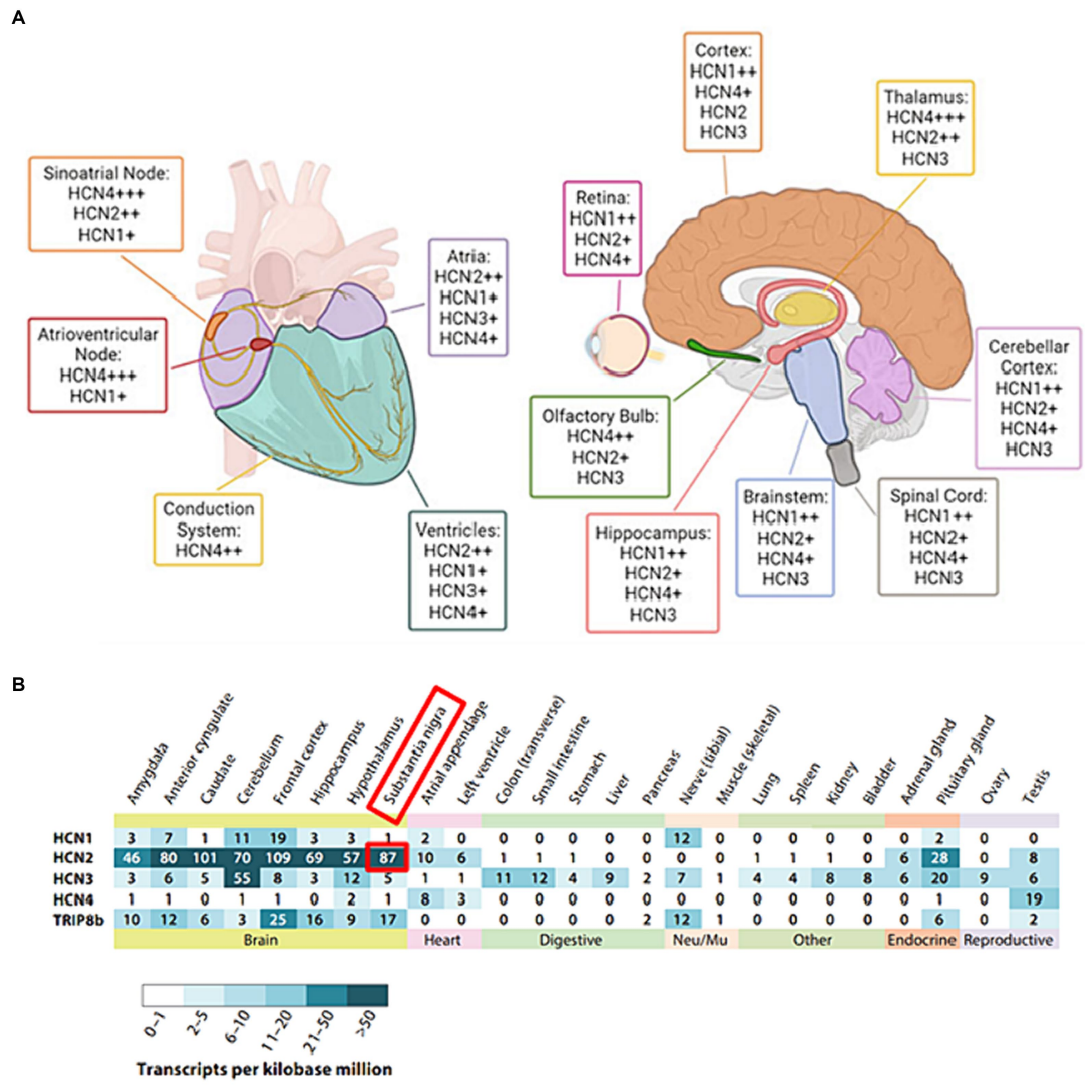
**(A)** The distribution of HCN isoforms in the heart and brain is shown. Distinct colors are used to represent particular locations in the heart (left) and brain (right) where distinct HCN isoforms are expressed. HCN4 is the predominant isoform in the sinoatrial and atrioventricular nodes of the heart. HCN1, HCN2, and HCN4 are abundant in the brain, with different distributions. The sign “+” denotes the relative abundance of certain isoforms. Adapted from Baruscotti et al. (2022) © 2022 European Biophysical Societies’ Association (EBSA). **(B)** Tissue RNA sequencing was used to examine the expression of HCN subunits in diverse adult human tissue samples. Adapted from Santoro and Shah (2020). © 2020 by Annual Reviews. All rights reserved (https://marketplace.copyright.com/rs-ui-web/mp/advanced-search/article/result). The investigation made use of Transcripts per Kilobase Million (TPM) data collected from GTEx Project tissue samples. The study included only tissue RNA expression data that were supported by findings from the Human Protein Atlas and/or the FANTOM5 data sets. The following URLs provide access to these additional datasets: GTEx Project (https://gtexportal.org), Human Protein Atlas (https://www.proteinatlas.org), and FANTOM5 (https://www.proteinatlas.org).

With a considerable degree of sequence homology, HCN1 and HCN2 isoforms show pronounced expression primarily within the central nervous system (CNS) (Figures 2A,B): HCN1 has a more restricted distribution and is found in specific regions such as the cortex and cerebellum (Figure 2B). HCN2, on the other hand, is more evenly distributed throughout the brain (Figure 2B). HCN1 and HCN2 channels are involved in various processes such as pace making activity, dendritic integration, and shaping of action potential firing patterns (Biel et al., 2009). HCN3 exhibits a wider expression pattern than other isoforms, being found in both CNS and peripheral tissues (Santoro and Shah, 2020). However, its specific functions are less well-characterized compared to the other isoforms, with potential contributions to sensory neuronal excitability and pain, necessitating further investigation for a comprehensive understanding of its functions (Lainez et al., 2019). HCN4 is abundantly found in the heart, chiefly in the sinoatrial node, and it is essential for producing the pacemaker currents (Baruscotti et al., 2022). Its unique expression and functional properties make HCN4 an essential player in the regulation of cardiac rate and rhythm (Baruscotti et al., 2022).

Furthermore, recent investigations have demonstrated that HCN channels can form both homomeric and heteromeric tetramers (Ulens and Tytgat, 2001; Much et al., 2003). For example, the co-assembly of HCN1 with HCN2 subunits activates faster than HCN2 alone but slower than HCN1 alone (Ulens and Tytgat, 2001). The voltage activation and cAMP sensitivity of such co-assemblies were found to be intermediate between those of individual subunits, thereby allowing for a spectrum of kinetic and voltage-cAMP properties (Much et al., 2003). *In vivo* studies have revealed the existence of functional heteromeric HCN channels in the form of HCN1/HCN2 heteromers in the mouse brain, suggesting their physiological significance (Much et al., 2003). Notably, N-linked glycosylation is essential for the efficient trafficking of channels to the plasma membrane, and heteromeric channels can compensate for glycosylation-deficient subunits, indicating more intricate regulation of these channels (Much et al., 2003). These findings suggest that heteromerization is critical in diversifying electrical transmission across the nervous system and the heart.

## 3 Known function and localization of HCN channels in the retina

Considering the various roles HCN channels play in the CNS, it is plausible that they may also play a role in retinal function if they exist in the retina. Indeed, it is well known that all four isoforms of HCN channels (i.e., HCN1-4) are distinctively expressed in various classes of neurons across the vertebrate retinas (Moosmang et al., 2001; Müller et al., 2003; Ivanova and Müller, 2006; Cangiano et al., 2007; Barrow and Wu, 2009; Della Santina et al., 2010, 2012; Bernard et al., 2014; Hellmer et al., 2016). Although HCN channels have not been well explored in the human retinas, it is worth noting that some patients who were prescribed ivabradine which is an HCN inhibitor to lower their heart rate reported visual disturbances, mostly phosphene-like phenomena (Gargini et al., 1999; Borer et al., 2003; Mao et al., 2003; Camm, 2006; Cangiano et al., 2007; Cervetto et al., 2007; Della Santina et al., 2010), indirectly suggesting the considerable expression of HCN channels in the human visual pathway. Because HCN4 channels support the I_f_ pacemaker currents in the SANs, the effectiveness of multiple HCN4 blockers in treating various cardiac problems has been thoroughly researched (Nakashima et al., 2021). It has been revealed that HCN channel malfunction is linked to a number of cardiac conditions including atrial fibrillation, atrioventricular block, ventricular tachycardia, and sinus node dysfunction (DiFrancesco, 2015). In addition, heart failure, ischemic cardiomyopathy, and hypertrophy have all been linked to a surge in ventricular HCN current (Cerbai et al., 1997; Hoppe et al., 1998; Stilli et al., 2001).

Numerous studies measured I_h_ current in retinal neurons to examine the role of HCN channels in the retina (Müller et al., 2003; Ivanova and Müller, 2006; Cangiano et al., 2007; Della Santina et al., 2012; Hellmer et al., 2016). Although the primary role of HCN channels is known to be ‘*generation of rhythmic potentials*’ in the CNS, PNS, or cardiac cells, it seems like their functions are somewhat different in the retina. For example, retinal HCN channels regulate (i.e., prevent) the membrane potential (V_m_) fluctuation in response to light stimuli (Della Santina et al., 2012), resulting in delayed b-wave of the ERG responses in HCN1-or HCN2-deficient mice (Della Santina et al., 2012). Also, it has been reported that I_h_ quickens the recovery of the V_m_ of rod bipolar cells (RBCs) to the baseline (Cangiano et al., 2007). They also observed a frequency-tuning property of I_h_ current by conducting whole-cell patch clamping and theoretical modeling for the cases with/without a pharmacological blocker of HCN (ZD7288). In more detail, the band-pass property of RBC impedance was changed to a low-pass filter profile after the pharmacological blockade of I_h_. Consistent with these results, after the inhibition of I_h_ current by injecting ivabradine into mice, the frequency response curve (FRC) of electroretinogram (ERG) was converted from the band-to low-pass shape (Della Santina et al., 2012). Since low-frequency contains a significant component of noise, the changed filtering characteristic allows the retina to send the noisy signal to the visual cortex, resulting in visual disturbances such as phosphenes (Della Santina et al., 2010). However, it is important to note that these pharmacological effects were reversible: specifically, both short-term (several hours) and long-term (3 weeks) administration of ivabradine inhibited the band-pass filter profile of the FRC in rat retinas, but these functions were all restored once the drug injection was stopped (Della Santina et al., 2010). Nonetheless, the specific functions of HCN3 and HCN4 isoforms are still not fully understood. Due to limited research conducted on them in the retina.

It is necessary to investigate how the expression of each HCN subtype varies across the retinal layers for a better understanding of their functions/characteristics in the retina. The localization of HCN channel isoforms has been precisely examined by several methods including fluorescence or electron microscopic immunochemistry (Demontis et al., 2002; Kim et al., 2003; Müller et al., 2003; Fyk-Kolodziej and Pourcho, 2007) and *in situ* hybridization (Moosmang et al., 2001). Table 1 summarizes the known distribution patterns of HCN isoforms across several species. Overall, the expression locations of HCN isoforms have not been well investigated in other species. In particular, HCN2-4 subtypes have received less attention while HCN1 channels have been relatively better studied. In all species but nonhuman primates, HCN1 isoform has been observed primarily in the photoreceptor inner segments (IS) but their distribution pattern appears distinct in each species. For example, in rodents, HCN1 isoform was also found throughout the whole retinal layers such as the nerve fiber layer (NFL), ganglion cell layer (GCL), inner plexiform layer (IPL), outer plexiform layer (OPL), and outer nuclear layer (ONL) (Table 1). Although rabbits showed the rodent-like distribution of HCN1 channel in the GCL, IPL, INL, and ONL (Kim et al., 2003), in chicken embryo retina, HCN1 isoform was only observed in IPL and OPL (Bernard et al., 2014). On the other hand, HCN1 isoform was expressed only in ONL and IPL of salamander (Barrow and Wu, 2009) and macaque (Puthussery et al., 2013) retinas, respectively. Other than HCN1, HCN2-4 isoforms were hardly explored, only in salamander retina, HCN3 channels were additionally reported in the IPL, OPL, and ONL (Barrow and Wu, 2009).

**Table 1:** Localization of HCN channels in the retina across different species.

**Retinal layers**		**NFL**	**GCL**	**IPL**	**INL**	**OPL**	**ONL**
Mouse	HCN1	Knop et al. (2008)	Moosmang et al. (2001) and Knop et al. (2008)	Cangiano et al. (2007), Knop et al. (2008), Della Santina et al. (2012), and Hellmer et al. (2016)	Moosmang et al. (2001) and Knop et al. (2008)	Cangiano et al. (2007) and Knop et al. (2008)	Moosmang et al. (2001), Cangiano et al. (2007), Knop et al. (2008), and Della Santina et al. (2012)
	HCN2		Moosmang et al. (2001)	Cangiano et al. (2007) and Della Santina et al. (2012)	Moosmang et al. (2001)	Cangiano et al. (2007) and Della Santina et al. (2012)	
	HCN3		Moosmang et al. (2001)		Moosmang et al. (2001)		
	HCN4			Hellmer et al. (2016)	Hellmer et al. (2016)	Hellmer et al. (2016)	
Rat	HCN1	Müller et al. (2003)	Müller et al. (2003), Fyk-Kolodziej and Pourcho (2007), and Stradleigh et al. (2011)	Müller et al. (2003), Fyk-Kolodziej and Pourcho (2007), and Della Santina et al. (2010)	Müller et al. (2003), Ivanova and Müller (2006), and Fyk-Kolodziej and Pourcho (2007)	Müller et al. (2003), Fyk-Kolodziej and Pourcho (2007), and Della Santina et al. (2010)	Müller et al. (2003), Fyk-Kolodziej and Pourcho (2007), and Della Santina et al. (2010)
	HCN2			Müller et al. (2003), Ivanova and Müller (2006), and Fyk-Kolodziej and Pourcho (2007)	Müller et al. (2003)	Müller et al. (2003), Fyk-Kolodziej and Pourcho (2007), and Della Santina et al. (2010)	Müller et al. (2003) and Fyk-Kolodziej and Pourcho (2007)
	HCN3			Müller et al. (2003)		Müller et al. (2003)	
	HCN4		Müller et al. (2003), Oi et al. (2008), Stradleigh et al. (2011), and Partida et al. (2012)	Müller et al. (2003) and Fyk-Kolodziej and Pourcho (2007)	Müller et al. (2003), Ivanova and Müller (2006), and Fyk-Kolodziej and Pourcho (2007)	Fyk-Kolodziej and Pourcho (2007)	
Rabbit	HCN1		Kim et al. (2003)	Kim et al. (2003)	Kim et al. (2003)	Kim et al. (2003)	Kim et al. (2003)
Chicken embryo	HCN1			Bernard et al. (2014)		Bernard et al. (2014)	
Salamander	HCN1						Barrow and Wu (2009)
	HCN3			Barrow and Wu (2009)		Barrow and Wu (2009)	Barrow and Wu (2009)
Macaque	HCN1			Puthussery et al. (2013)			

The outer nuclear layer (ONL) includes photoreceptor inner segments. HCN1 channels are widely distributed in all layers except nerve fiber layer (NFL) in mouse and rat retinas. HCN2 channels are expressed in more confined manner in inner plexiform layer (IPL) and outer plexiform layer (OPL) in both mouse and rat retinas. The expression pattern of HCN3 is not consistent across species. In inner nuclear layer (INL) and ganglion cell layer (GCL) of rat retinas, HCN4 channels are mostly expressed.

Even with the complexity of their distribution in plexiform layers (i.e., OPL and IPL), the unique localization of each HCN isoform is somewhat revealed. For instance, HCN1 was observed not in rod spherules at OPL but in cone pedicles (Fyk-Kolodziej and Pourcho, 2007), as well as densely packed in the middle of IPL. HCN2 subunits were found in the dendrites of RBC as dotted patterns at OPL and clustered at ribbon synapses in the mouse retina (Cangiano et al., 2007); in contrast, HCN2 subunits were found at the axon terminals of ON type bipolar cells, especially type 8 and RBC in the rat retina (Müller et al., 2003). HCN3 was mainly concentrated presynaptically in cone pedicles (Müller et al., 2003). HCN4 showed strong immunochemistry responses at the axon terminal of type 3 and 5 bipolar cells in the IPL (Müller et al., 2003; Fyk-Kolodziej and Pourcho, 2007). However, it seems the co-localization of HCN isoforms at the same strata and expression of HCN channels in amacrine and horizontal cells further complicates the precise investigation of their localization.

The unique HCN distribution patterns across the retinal layers seems to be because each HCN isoform carries different functions. Since HCN channels are primarily concentrated at synaptic terminals, it is possible that they are involved in neurotransmitter release to regulate visual perception including both ON and OFF responses (Müller et al., 2003). To clarify this point, HCN channels need to be co-stained with various markers that can show the neurotransmitter receptor region, such as mGluR6 for ON-bipolar cells, GluR1 for OFF-bipolar cells, and CabP for horizontal cells (Müller et al., 2003). Furthermore, the cell-type-dependent localization accuracy can be enhanced by introducing specific retinal neuron markers such as ChAT (Voigt, 1986) for cholinergic amacrine cells, recoverin for cone bipolar cell types 2 and 8 (Milam et al., 1993), and vesicular glutamate transporter 1 (VGluT1) for OPL and IPL (Mimura et al., 2002; Johnson et al., 2003; Fyk-Kolodziej et al., 2004; Gong et al., 2006). These specific markers will help better understand about more specific location of each HCN channel and explore their unique function.

## 4 Known functions of HCN channels in the central nervous system other than the retina

Comprehensive understanding of the roles of HCN channels existing in other parts of the central nervous system (Baruscotti et al., 2010; Santoro and Shah, 2020) may help us speculate unknown role(s) of HCN channels expressed in the retina. The well-known roles of HCN channels are (1) assisting dendritic integration, (2) creating neural oscillation, and (3) regulating rhythmic activity (Benarroch, 2013). First, HCN channels participate in dendritic integration (Chu and Zhen, 2010): since a single excitatory postsynaptic potential (EPSP) is too small to generate action potentials (APs), the integration of EPSPs in dendrites is typically necessary (Magee, 2000). HCN channels reduce membrane resistance, followed by an outward K^+^ current, enabling EPSP amplitude decrement (Magee, 2000; Benarroch, 2013). Also, the heterogeneous distribution of HCN channels in a single neuron provides location-independent EPSP shaping. In the CA1 pyramidal neuron, for example, more HCN1 channels are located in distal than proximal dendrites. Furthermore, HCN1 is abundant in dendrites, whereas HCN2 is predominant in soma. The heterogeneous distribution of HCN channels provides an equivalent intensity of EPSP regardless of their location in a given cell (Berger et al., 2003; Benarroch, 2013).

Second, HCN channels are essential for oscillations in both individual cells and cellular networks. A prime example of this can be observed in thalamocortical neurons, where two distinct depolarization modes exist: the transmission mode and the burst mode (Benarroch, 2013; Deutsch et al., 2021). Sensory inputs depolarize thalamocortical neurons, generating single spikes that appear as delta waves in electroencephalography (EEG) during wakefulness and non-rapid eye movement (non-REM) sleep (Benarroch, 2013; Maki-Marttunen and Maki-Marttunen, 2022). The combination of the I_h_ current and low-threshold Ca^2+^ current (T-current) during hyperpolarization of thalamocortical neurons results in a burst mode (Benarroch, 2013; Maki-Marttunen and Maki-Marttunen, 2022). In a burst mode, HCN channels are activated when thalamocortical neurons are hyperpolarized, generating I_h_ current until T-channel-based rebound spikes appear (Benarroch, 2013; Maki-Marttunen and Maki-Marttunen, 2022). The depolarized membrane from rebound spikes inhibits both HCN and T-channels, leading to a hyperpolarized membrane (Benarroch, 2013; Maki-Marttunen and Maki-Marttunen, 2022). The hyperpolarized membrane then goes through the first step of the burst mode cycle again for single-cell oscillation, activating cortical neurons and a widespread area of the brain (Benarroch, 2013; Maki-Marttunen and Maki-Marttunen, 2022). HCN channels also participate in the production of oscillations in not only single cells but also neural networks. The hyperpolarization of GABAergic thalamic reticular neurons (TRNs) is caused by afferent inputs, which generate a burst mode of TRNs (Benarroch, 2013). Bursting TRNs produce rhythmic inhibitory postsynaptic potentials (IPSP), which hyperpolarize thalamocortical neurons. Hyperpolarized TRNs generate I_h_ current that can initiate Ca^2+^ rebound spikes (Benarroch, 2013). Depolarization caused by T-currents activates both TRNs and cortical pyramidal neurons, generating synchronization between the cortex and the thalamus. The oscillation of cortical and thalamic networks produces spindle waves in the cortex (Berger et al., 2003; Benarroch, 2013; Watari et al., 2013; Wahl-Schott et al., 2014), which are known to be a distinctive feature known to have numerous sleep-related functions such as memory consolidation and cortical development (Andrillon et al., 2011).

Lastly, rhythmic activity is regulated by HCN channels especially in the brain and the heart. There are two ways to induce the rhythmic electric signals in these organs: one is controlling the retention time from the hyperpolarization state, and the other is inducing oscillation of single cell and/or cellular network. HCN channels regulate the heart rate by controlling hyperpolarization retention time. It is well known that HCN channels modulate the cardiac rhythm in sinoatrial node (SAN) cells which produce self-triggered action potentials (AP) using a network of interconnected oscillators, and their collective output serves as the starting point for each regular heartbeat (MacDonald et al., 2020). When an action potential fires, the membrane potential reaches a hyperpolarization state after it passes the peak of the amplitude and repolarization state due to slow kinetics of voltage-gated K^+^ channels. Then, HCN channels are activated to induce return to the RMP (Berger et al., 2003; Benarroch, 2013). The rapid return by HCN channels is important for controlling the heart rate. The administration of ivabradine, known as a blocker of HCN channels, causes deceleration of heart rate because the duration of the hyperpolarization state becomes longer due to the membrane retainment caused by HCN channel suppression (Tse and Mazzola, 2015).

## 5 Disorders related to HCN channels

Given the recent report that revealed the contribution of HCN1 isoform in the degeneration of photoreceptor cells (Schön et al., 2016), a portion of retinal degenerative diseases may be somewhat linked with malfunction of HCN channels. There are several studies suggesting a possibility that HCN channels could be an attractive therapeutic target for some neurological disorders ranging from epilepsy to Parkinson’s disease (Kase and Imoto, 2012; DiFrancesco and DiFrancesco, 2015; Ramirez et al., 2018). For example, researchers have speculated that the dysfunctional association of HCN channels with the pathogenesis of epilepsy is due to their role in modulating neuronal excitability (Baruscotti et al., 2010). A study using a knock-out (K/O) mouse model has revealed the association between changes in HCN channel function and epileptogenesis. For instance, HCN1 K/O in mice resulted in elevated cortical excitability due to the loss of function (LOF) in HCN1 channels, leading to epileptogenesis (Huang et al., 2009). Another investigation revealed increased prevalence of spontaneous absence seizures in global HCN2 channel K/O mice (Ludwig et al., 2003). In both aged macaques and in humans with Alzheimer’s disease, amounts of HCN1 channels were found to be lowered in the temporal lobe (Saito et al., 2012). This specific study suggests that the increased susceptibility to epileptic seizures in Alzheimer’s disease patients may be due to the downregulated HCN1 channels (Saito et al., 2012).

The exact mechanisms underlying how dysfunctional HCN channels contribute to epilepsy remain still unclear, but there are several hypotheses based on previous studies: one theory suggests that abnormal HCN channel function may upset the balance of neuronal excitability (Kessi et al., 2022). HCN channels regulate the RMP of neurons, which represents the amount of electrical charges across the cell membrane when the neuron is not receiving any input (Shah, 2014). It has been known that, in individuals with epilepsy, there are often imbalances between excitatory and inhibitory neurotransmission, causing neurons to become overactive and more prone to abnormal firing (Shao et al., 2019). Since HCN channels are essential for controlling the pacemaking activity of particular neurons, which is crucial for coordinating the activity of large groups of neurons, disrupted HCN channel function may also disrupt normal neuronal firing patterns in the brain (Maccaferri and McBain, 1996). Therefore, disturbances in this activity can lead to seizures. The effects of malfunctioning HCN channels on neuronal excitability, firing patterns, and pacemaker activity have been linked to the onset and disease persistence of epilepsy (Kase and Imoto, 2012; Kessi et al., 2022).

It has been reported that Parkinson’s disease (PD) has something to do with HCN channels. Interestingly, the midbrain’s substantia nigra pars compacta (SNc) dopaminergic neurons have the greatest levels of HCN2 and HCN4 expression (Figure 2B) among all HCN isoforms (Notomi and Shigemoto, 2004). A previous study on animal models of PD has revealed that HCN channel downregulation occurs gradually after the depletion of dopamine and the loss of dopaminergic neurons, while the protein expression level of the other channels remains unchanged (Good et al., 2011). In MitoPark mice, a well-established animal model of PD, a reduction in the HCN current density in SNc dopaminergic neurons was observed, followed by the initiation of epileptic seizures (Good et al., 2011). Moreover, recent research has reported some evidence of a relationship between PD pathogenesis in humans and dysfunctional HCN channels by analyzing electrophysiological data obtained from models treated with the toxic chemical 1-methyl-4-phenylpyridinium (MPP+), which can induce PD-like selective degeneration of nigral dopaminergic neurons, suggesting that HCN blockage from MPP+ treatment may increase synaptic excitability (Masi et al., 2013).

Several mechanisms are suggested for the association between HCN channels and PD: one mechanism is related to the modulation of neuronal excitability (Chang et al., 2019). The HCN channels in dopaminergic neurons of SNc are responsible for controlling firing rate and pacemaker activity, and their malfunction can lead to reduced excitability, resulting in decreased dopamine release and contributing to PD (Chu and Zhen, 2010). Additionally, HCN channel dysfunction can disrupt the basal ganglia circuitry, which controls movement (Chang et al., 2019). Unintentional motor movement is a hallmark of PD and is known to be caused by the degeneration of substantia nigral dopaminergic neurons of the brain (Kalia and Lang, 2015). Thus, HCN channel malfunction in dopaminergic neurons can lead to abnormal movement patterns due to the disruption of pacemaker activity and synchronization of basal ganglia circuitry (Chang et al., 2019). Another mechanism causing PD is that malfunction of HCN channels in dopaminergic neurons might result in an increase in oxidative stress and the formation of reactive oxygen species (Chang et al., 2019). Cellular components, including proteins and lipids, can be destroyed by this oxidative stress, leading to neuronal dysfunction and degeneration and contributing to PD.

HCN channels play a vital part not only in neurogenerative diseases but also in both inflammatory and neuropathic pain by initiating and regulating the firing rate of APs responsible for pain (Jiang et al., 2008). For instance, a recent study selectively eliminated HCN2 in the Na_V_1.8 sensory neurons to investigate the function of HCN2 isoform in pain generation and persistence (Emery et al., 2011, 2012). In contrast to how HCN2 K/O mice responded to acute pain in the absence of inflammation, their findings demonstrated that these mice did not experience pain in response to diverse inflammatory stimuli (Emery et al., 2011, 2012). HCN channels have been discovered to be involved in the experience of pain brought on by direct nerve damage, as evidenced by the finding that subtype-independent pharmacological blockage of HCN channels reduced neuropathic pain (Ding et al., 2016). Also, the removal of HCN1 gene resulted in partial reduction of neuropathic pain caused by nerve damage (Momin et al., 2008).

In addition to their neurophysiological roles, HCN channels seem to have a vital role in the cellular life cycle by affecting both the cell growth and cell death mechanisms. According to a recent study (Nordstrom et al., 2022), it was reported that HCN channels affect the differentiation and migration of neural progenitor cells: HCN channel knock-out (K/O) cell lines showed increased hypoxia affection and decreased differentiation and migration in neurospheres. In another study, Yeh et al. found a decreased expression level of HCN channels in aging oocytes, and suggested that the channels may be involved in the growth and differentiation of ovarian follicles (Yeh et al., 2008). Also, HCN channels have something to do with cell death: it has been revealed that the influx of Ca^2+^ through HCN2 channels leads to apoptosis in the primary culture of cortical neurons and in pulmonary carcinoma cells, and the down-regulation of HCN2 stopped this process (Norberg et al., 2010). However, comprehensive cell viability studies regarding HCN channels are still lacking and its underlying mechanism(s) remain largely unknown.

## 6 Conclusion

The passage of Na^+^ and K^+^ ions across cellular membranes is controlled not only by Na^+^/ K^+^ channels but also by HCN channels which need to be considered as significant membrane proteins (Benarroch, 2013; Sartiani et al., 2017). They maintain the RMP, regulate the firing of EPSPs, contribute to dendritic integration, and generate oscillations in neural networks (Biel et al., 2009; Chang et al., 2019). The heterogeneous distribution of HCN channel isoforms enables efficient summation of EPSPs (Berger et al., 2003; Benarroch, 2013). Neurological conditions including epilepsy and PD have been related to dysfunctional HCN channels that impact basal ganglia circuitry, neuronal firing patterns, and neuronal excitability (Kase and Imoto, 2012; Masi et al., 2013; Chang et al., 2019; Kessi et al., 2022). For the purpose of creating novel therapies for these disorders, it seems critical to fully comprehend the function and control of HCN channels.

In the context of the retina, HCN channels have not been thoroughly studied. For example, previous studies revealed that different HCN isoforms have distinct localization patterns but mostly in the rodent retinas. Although each HCN subtype seems to unique function in visual processing, it still lacks comprehensive understandings for all four subtypes. For instance, it has been reported HCN1 channels modulate photoreceptor responses and signal transmission in the outer retina (Seeliger et al., 2011; Della Santina et al., 2012) and HCN2 channels regulate the output of bipolar and ganglion cells (Cangiano et al., 2007); but functions of HCN3 and HCN4 subtypes have not been explored. Also, it is important to note that the distribution patterns of four HCN isoforms remain unknown in the primate retinas, highlighting the need for additional studies to comprehensively understand the functional roles of HCN channels in natural viewing of humans. The thorough understanding of HCN channels may guide targeted interventions and exploring their interactions with other retinal proteins and signaling pathways, ultimately advancing our knowledge about the mechanism of action in both healthy and pathologic retinas.

## Author contributions

DK: Investigation, Visualization, Writing – original draft, Writing – review & editing. HR: Investigation, Visualization, Writing – original draft, Writing – review & editing. H-ML: Investigation, Writing – original draft, Writing – review & editing. SK: Investigation, Writing – original draft, Writing – review & editing. MI: Conceptualization, Funding acquisition, Investigation, Methodology, Project administration, Supervision, Writing – original draft, Writing – review & editing.
