# Corrigendum: Localization of hyperpolarization-activated cyclic nucleotide-gated channels in the vertebrate retinas across species and their physiological roles

**DOI:** 10.3389/fnana.2024.1445452

**Published:** 2024-06-20

**Authors:** Daniel Kim, Hyeonhee Roh, Hyung-Min Lee, Sang Jeong Kim, Maesoon Im

**Affiliations:** ^1^Brain Science Institute, Korea Institute of Science and Technology (KIST), Seoul, Republic of Korea; ^2^Department of Biomedical Sciences, College of Medicine, Seoul National University (SNU), Seoul, Republic of Korea; ^3^School of Electrical Engineering, College of Engineering, Korea University, Seoul, Republic of Korea; ^4^Division of Bio-Medical Science & Technology, KIST School, University of Science & Technology (UST), Seoul, Republic of Korea; ^5^KHU-KIST Department of Converging Science and Technology, Kyung Hee University, Seoul, Republic of Korea

**Keywords:** HCN channels, central nervous system (CNS), retina, localization across species, retinal degeneration

In the published article, there was an error in the affiliation of Hyeonhee Roh. Instead of “**Hyeonhee Roh**^**1,3,†**^”, it should be “**Hyeonhee Roh**^**3†**^”.

The authors apologize for this error and state that this does not change the scientific conclusions of the article in any way. The original article has been updated.

